# Anatomical Correlates to Nectar Feeding among the Strepsirrhines of Madagascar: Implications for Interpreting the Fossil Record

**DOI:** 10.1155/2011/378431

**Published:** 2011-10-17

**Authors:** Magdalena N. Muchlinski, Jonathan M. G. Perry

**Affiliations:** ^1^Department of Anatomy and Neurobiology, College of Medicine, MN210 Chandler Medical Center, University of Kentucky, Lexington, KY 40536, USA; ^2^Department of Anatomy, Midwestern University, Downers Grove, IL 60515, USA

## Abstract

One possible ecological scenario for the origin of primates is the archaic pollination and coevolution hypothesis. Its proponents contend that the consumption of nectar by some early primates and the resulting cross-pollination is an example of coevolution that drove adaptive radiations in some primates. This hypothesis is perhaps ecologically sound, but it lacks the morphology-behavior links that would allow us to test it using the fossil record. Here we attempt to identify cranial adaptations to nectar feeding among the strepsirrhines of Madagascar in order to provide such links. Many Malagasy strepsirrhines are considered effective cross-pollinators of the flowers they feed from, and nectar consumption represents as much as 75% of total feeding time. Previous studies identified skeletal correlates to nectar feeding in the crania of nonprimate mammals; from these, nine cranial measurements were chosen to be the focus of the present study. Results indicate that *Cheirogaleus, Varecia*, and *Eulemur* mirror other nectar-feeding mammals in having elongated crania and/or muzzles. These strepsirrhines might be effective cross-pollinators, lending support to the coevolution hypothesis.

## 1. Introduction

Several traits distinguish primates from other mammals, extant and extinct. Foremost among these are orbital convergence, divergent first digits, and the possession of flattened nails instead of claws (at least on the hallux and pollex) [1–4]. The consensus of decades of debate appears to be that this suite of traits evolved as a means to more effectively forage in the small-diameter, terminal branches of trees (perhaps especially angiosperms) at night [1–4]. However, there is still disagreement about the object of the foraging activities. Cartmill [2–4] proposed that the earliest true primates were hunting insects and that convergent orbits were useful not only in guiding locomotion in the fine-branch environment but also in guiding the limbs as the primate seizes its mobile prey. Since its initial publication, the visual predation hypothesis of primate origins has received substantial support from many different lines of evidence [3, 5, 6]. Nevertheless, competing hypotheses exist and some are ecologically compelling.

Sussman and Raven [7] proposed that, although early primates may have been looking for insects in the terminal branches, they were also pursuing fruits, flowers, and especially nectar. This model stresses the symbiotic relationship between fruits, flowers, and insects, as well as the primates that were attracted to them. The evidence for the model includes the observation that nectar-feeding primates are present where there are angiosperms that produce large, tough, odorous, drab flowers with copious nectar and where there are also few species of nectar-feeding bats. This is also true of environments where nectar-feeding marsupials thrive [8]. Thus today, most nectar-feeding primates live in Madagascar. It is possible that nectivory was common in early primates, but, outside Madagascar, nectivorous bats largely replaced these nectivorous primates. 

Unlike the nocturnal visual predation hypothesis, the angiosperm coevolution hypothesis lacks strong anatomical backing. The objective of this study is to determine if the anatomical adaptations for nondestructive nectar feeding, as identified in other mammalian orders, are found in nectivorous Malagasy strepsirrhines. Results of this work could provide the needed anatomical evidence to test Sussman and Raven's [7] hypothesis using the fossil record.

There are currently two definitions to describe this form of interactive evolution: coevolution and diffuse coevolution. Coevolution is reciprocal evolution. In strict coevolution, there is a change in the genetic composition of one species in response to a genetic change in another [9–14]. Diffuse coevolution, however, includes the selective pressures exerted between one broad animal taxon and one broad plant taxon, which are linked by mutualistic interactions (e.g., cryptic Figs and M/L cone polymorphism among primates [15]) [12, 16, 17]. Diffuse coevolution may explain many of the anatomical specializations seen in primates today. Because we are unlikely to detect true cases of coevolution in the fossil record, we will consider mainly the concept of diffuse coevolution for the remainder of this study.

Primates can diffusely coevolve and strictly coevolve with angiosperms. These two mutually beneficial interactions are nectar feeding/cross-pollination and fruit eating/seed dispersal. Anthropologists studying coevolutionary processes generally focus their attention on fruit eating and seed dispersal [18, 19]. Exclusive coevolution between a single plant species and a single vertebrate species is unlikely to occur within such a complex and multi-perpetrator interaction as frugivory [9, 18, 20, 21]. Fruiting trees do not receive an immediate reward from the dispersers because there is no guarantee that the seeds will (1) be dispersed and (2) germinate [22, 23]. 

Nectar feeding/cross-pollination is a more likely example of a true coevolved relationship. Nectar produces immediate rewards for both the animal and the plant [24, 25]. Nectar feeding is a two-step, reciprocal coevolutionary process. Flowers produce nectar to attract potential cross-pollinators. When a mammal feeds on nectar, pollen accumulates on hair of the face and muzzle. Immediately rewarded with nectar, the animal is enticed to visit more nectar-producing plants of the same species, in turn cross-pollinating each new flower they visit. Additionally, nectar-feeding is more likely to be seen in the fossil record, since extant cross-pollinators have a suite of anatomical features associated with a reduction in jaw robusticity and muscle attachment sites (likely accompanied by reduction in jaw muscle size). 

Primate nectar feeding and cross-pollination of flowers is well acknowledged today [7, 26–33]. The strongest evidence of cross-pollination is documented among the strepsirrhines of Madagascar. Nectar appears to be the second most important food item in the annual diets of most Malagasy strepsirrhines [34] and provides substantial nutrition (proteins, sugars, and seven essential amino acids) for the whole troop [29, 35–39]. Malagasy strepsirrhines that regularly or seasonally feed on nectar are *Cheirogaleus major*, *Microcebus rufus* [40], *Eulemur mongoz* [7, 32, 41, 42], *Eulemur rubriventer* [34, 43]*, Eulemur fulvus rufus *[34, 44, 45], *Eulemur fulvus sanfordi, Eulemur coronatus *[34],* Eulemur macaco *[45–47], and* Varecia variegata variegata* [36, 37]. *Eulemur mongoz mongoz* has been observed spending 80% of its foraging time feeding from the nectar-producing parts of four species of plants [7, 42, 48]. Eighty percent of this time was spent on the nectar of the kapok tree (*Ceiba pentandra*) [7, 48]. Similar feeding behaviors are observed year-round for *Eulemur mongoz*. *Varecia variegata variegata* spends approximately 25% of its feeding time exploiting nectar, making nectar the second most important food item in its diet [36, 37]. However, during periods of fruit scarcity, the Traveler's Palm (*Ravenala madagascariensis*) is in bloom. During this period, over 72% of *Varecia*'s feeding time is spent on this one plant; thus *Ravenala* is *Varecia*'s primary source of caloric intake [36]. It is important to note that neither *Eulemur mongoz* nor *Varecia variegata* is destructive to any part of the plant. Nondestructive nectar feeding, when combined with the morphology of some “lemur-loving” plants (e.g., large durable flowers) [36, 37], implies a possible mutualism between certain Malagasy strepsirrhines and Malagasy plants. The circumstantial evidence suggests that many Malagasy plant species may actually depend on animals as important cross-pollinators [31, 32, 36, 37, 48]. 

Here we propose that *Eulemur*, *Lepilemur*, *Microcebus*, *Phaner*, *Varecia*, and possibly *Cheirogaleus *can be considered cross-pollinators. The monophyly of strepsirrhines offers an excellent opportunity to study adaptations in skull shape and tooth morphology in potential response to diet, given that we find frugivores, folivores, insectivores, and nectivores in the group. Different adaptive pressures, unique flora and fauna, and the abundance of vacant niches have allowed this group to diversify in relative isolation.

### 1.1. Anatomical Modifications for Nectar Feeding

Nectivorous marsupials and bats have several anatomical modifications that assist in nectar feeding [38, 49–51]. Nonprimate nectar feeders generally have one or all of the following traits: small conical projections on the tongue that appear feathered or ribboned, laterally scaled muzzle hair, the ability to see ultraviolet light, skeletal modifications such as an elongated snout, and/or diminutive dentition. These traits promote nondestructive nectar feeding and cross-pollination.

Several Malagasy strepsirrhines appear to parallel nectar-feeding bats and marsupials in their soft-tissue anatomy. Convergent with many other nectar-feeding mammals, *Eulemur rubriventer* [38], *Allocebus trichotis *[52], and *Varecia variegata* [53] are documented to have brush-like, feathered, or ribboned tongues. These modifications are purported to increase nectar uptake while feeding [38]. Moreover, the Malagasy strepsirrhines *Varecia variegata*,* Eulemur mongoz*, and *Microcebus murinus* have laterally scaled muzzle hairs [54]. Hair is described as laterally scaled when the outermost layer, the cuticle plates/scales, attach to the innermost layer (medulla and cortex) at an angle (e.g., cuticle attaches at a 45 degree angle relative to the cortex). Laterally scaled hair is proposed to aid in cross-pollination of flowers because more pollen can be captured in these hairs than in regular hair or fur [51, 55]. 

In a craniometric study of 28 extant marsupials, bats, and primates, Dumont [49] demonstrated that it is possible to discriminate among fruit, gum, and nectar feeders with an 88–94% success rate. Nectar feeders have significantly longer skulls and dentaries and relatively lower coronoid processes than fruit and exudate feeders [49]. Nectivores also have narrower skulls, perhaps representing a reduction in the masticatory system [49, 50]. 

No prior study has evaluated the skeletal anatomy of Malagasy strepsirrhines for nectar-feeding correlates. Malagasy strepsirrhines converge in many aspects of their soft-tissue anatomy and in many cases behaviorally with other nectar-feeding mammals. Based on these homologies, one can expect to see skeletal similarities. If there are cranial variables associated with nectivory among primates, then it will be possible to infer nectivory in the fossil record.

In general, we expect that the more important the nectar is in the diet, the more likely that a primate will avoid damaging the plant during feeding (e.g., destruction of the flower petals). Therefore, we can divide nectivores into two categories: those that are generally nondestructive to the flower (ND) and those that are destructive to the flower (D), with members of the former category expected to show more/greater adaptations toward nectivory. The nectivorous suite of adaptations is predicted to include features associated with muzzle elongation and narrowing (for greater access to nectaries with less risk of damage to surrounding structures). This condition is also expected to be associated with features that reflect masticatory strength reduction (e.g., decreased chewing muscle size and cross-sectional area) and a reduction in the need for gape (e.g., decreased chewing muscle fiber length). This assumes that a greater dependence on nectar implies less reliance on foods that are resistant to mastication or that require wide ingestive gapes.

## 2. Methods

### 2.1. Sample

Data were collected on 19 Malagasy strepsirrhine species representing four families: *Lemuridae, Cheirogaleidae, Lepilemuridae, Indriidae* (*N* = 250; Table 1). Using the recent literature, general dietary categories were assigned to each species in this sample (Table 1). Malagasy strepsirrhines traditionally have been categorized as frugivorous, folivorous, and/or insectivorous [56]. Nevertheless, all feed on at least some nectar. In this study, we put aside traditional categorizations and focus on the mode of nectar consumption (Table 1). In some species, the flowers are damaged and/or consumed, and we label those species as destructive flower feeders (D). In others, the animal appears to avoid damage to the plant while harvesting only nectar; these are labeled nondestructive (ND). Finally, some feed on nectar, but it is unknown whether or not they damage the plant in the process; these are labeled unknown (UN). Most of the nocturnal strepsirrhines (members of the family Cheirogaleidae) are reported to forage on flowers; however, whether these animals consume the flowers or just feed on nectar within them is still unclear [57, 58]. Thus, we labeled most small nocturnal strepsirrhines as unknown (UN).

### 2.2. Morphometric Variables and Predictions

**Figure 1:**
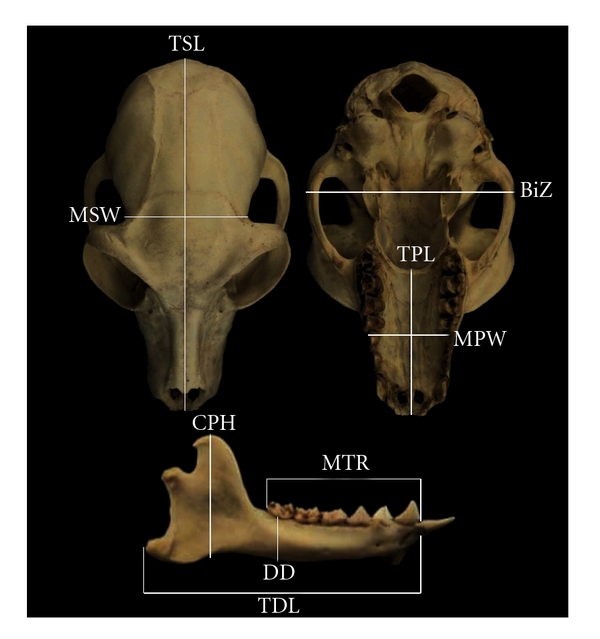
A photograph of a *Lemur catta* skull illustrating the osteometric points used to calculate the nine cranial measurements associated with nectivory in mammals. Coronoid process height (CPH): basal point on the angular process to the apex of the coronoid process. Palate width at M^1^ (MPW): the distance between the lateralmost point on the left M^1^ alveolus and the corresponding point on the right side. Minimum skull width at temporal fossa (MSW): breadth of cranium directly posterior to the postorbital bars. Bizygomatic width (BiZ): the greatest distance between the outer margins of the zygomatic arches, the distance between zygion points. Maximum tooth row length (MTR): the distance from the posterior edge of M_3_ to the anterior edge of the lower canine. Dentary depth at M_3_ (DD): the vertical distance from inferior margin of the mandible to the ectomolare of M_3_. Total dentary length (TDL): the distance from the posterior edge of the gonion to the alveolare (infradentale superius). Total palate length (TPL): the distance between staphylion and prosthion, which is sometimes called medial palatal length. Total skull length (TSL): the distance between akrokranion and prosthion. Akrokranion is the most aboral (nuchal) point on the vertex of the cranium. Two measurements not shown in this image are M_1_ length and width. Together these measurements were used to derive a crude measure of molar area.

Of 22 linear cranial variables studied, Dumont [49] selected nine traits that effectively discriminate nectar feeders from members of other dietary categories. Nectivores were found to have anatomical features associated with a reduction in masticatory strength, narrow and long palates, and relatively longer skulls than animals in other dietary categories. A description of each variable and its relationship to dietary preferences are detailed in the section (Figure 1).

#### 2.2.1. Cranial Variables

Minimum skull width is usually directly posterior to the postorbital bar in strepsirrhines. Therefore, a measurement of minimum skull width is generally reflective of the degree of postorbital constriction. When scaled to body size, this is a rough estimate of anterior temporalis thickness [50]. However, to more accurately estimate the thickness of the anterior part of the temporalis muscle, minimum skull width was subtracted from bizygomatic width. This reflects the fact that, in every strepsirrhine studied, the temporalis nearly fills the space between the lateral side of the cranium and the medial side of the zygomatic arch at the level of the zygomatic arch [67]. Dumont [49] reported that nectar feeders have relatively weaker masticatory muscles than non-nectar feeders, and; therefore, we predict that nondestructive nectar feeders will have relatively greater minimum skull width or a relatively thin temporalis muscle (bizygomatic width—postorbital constriction) than animals that must employ more masticatory force in food processing (Figure 1). 

In previous studies, nectar feeders demonstrated an increase in total skull length, total palate length, and total dentary length as well as a decrease in total palate width [49, 50, 68]. However, a concrete functional explanation has not been suggested for this pattern. Having a longer, narrower muzzle and skull reportedly aids in nondestructive nectar feeding by allowing the rostrum to penetrate more deeply into flower nectaries without affecting the surrounding flower petals [47, 48, 57]. Therefore, we expect that nondestructive nectar-feeding lemurs will have longer and narrower palates.

#### 2.2.2. Mandibular Variables

Mandible depth was taken at M_3_. Vertically deep mandibular corpora appear to be signal of adaptation to counter increased sagittal bending stress on the balancing side of the mandible during unilateral mastication [69, 70]. Nectar-feeding marsupials and bats have relatively shallower mandibles for their body size, perhaps as a result of diminished masticatory (and bending) loads [49]. We expect strepsirrhines that rely on nectar to a greater degree to have relatively shallower jaws than, for example, their more folivorous counterparts.

The coronoid process is the insertion site for the temporalis. Animals that have reduced masticatory strength tend to have relatively lower/shorter coronoid processes than expected for their body size [67]. We expect more specialized nectivorous strepsirrhines to have shorter coronoids than those that rely less on nectar [49]. We recognize that the height of the coronoid process might be affected, in principle, by adaptations for gape. Therefore, selection for a low coronoid because of overall masticatory reduction (in a nectivore) might be dampened by a reduction in the need for gape. However, it is unclear that coronoid height is directly affected by gape apart from its relationship to condyle height [71, 72]. Nevertheless, the comparative work on nectivores (that of Dumont) suggests that coronoid process height is related to the size (and insertion) of temporalis.

#### 2.2.3. Dental Variables

Two dental measurements were taken, upper second molar area (i.e., the product of the tooth's maximum mesiodistal and buccolingual diameters) and maximum tooth row (i.e., postcanine tooth row) length. These measurements were taken because animals with smaller molars and shorter postcanine teeth tend to have less folivorous diets [56, 73]. According to Dumont [47], nectar feeders and frugivores share a reduction in dentition; however, nectar feeders are reported to have relatively smaller teeth than their frugivorous counterparts. The most dedicated nectar feeders in this sample (i.e., nondestructive foraging ones) are predicted to have an overall reduction in molar area and postcanine tooth row length.

### 2.3. Sexual Dimorphism

Body weight data are not available for many of the museum specimens used in this study. Table 1 lists published species mean weights (kg) calculated as a surrogate for individual body weights [74]. The data collected for this study are characterized by unequal sample sizes both among species and between sexes within a species. The strepsirrhines of Madagascar are widely accepted as being monomorphic based on living body weights [74–76]. Morphological tests confirm that there were no significant differences between the sexes or between wild/captive osteological specimens. Therefore, to maintain variance, values for all individuals of a species were pooled to generate each species mean.

### 2.4. Statistics

Allometric analysis investigates the relationship between shape and body size. Regression analysis allowed us to identify associations between cranial morphology and diet across a wide range of body sizes, while accounting for the effects of body size variation on morphological variation. Reduced major axis (RMA) is the most appropriate regression technique used when error is expected of both the *X* (e.g., body mass) and *Y* (e.g., cranial length) variables equally; here, no assumption is made about the dependence of one variable on the other. Furthermore, we are not using the regression equations for prediction, but rather we are only interested in the biomechanical relationship between a cranial measurement and body size [77–79]. For the sake of comparison and completeness, both least squares and reduced major axis models are employed throughout this allometric investigation.

Body mass (kg) was selected as the “independent” variable for the allometric analysis. Some researchers prefer to employ palate length as a body size surrogate [80, 81]. However, palate length appears to have its own unique functional parameters that could vary independently of other cranial features [82]. Given that palate length has been described as an informative character for the identification of nectar feeders, palate length will not be used for any allometric analysis here. The data were not normally distributed, and; therefore, we transformed them into natural logs. Simple bivariate plots were then made for the natural log of each variable against the natural log of body mass. Confidence bands were places around each regression line. The 95% confidence band can be used to evaluate the allometry of the regression line and, thus, the scaling relationship between the two variables. We also tested to see if there were significant differences between the observed slope and the predicted slope of isometry. For the least squares regressions, analyses were calculated by hand following standard statistical procedures as outlined by Zar [83]. SMAT(R) was used to test for differences between the observed reduced major axis regression slope and that of a hypothesized slope. Table 2 lists the RMA and the LS regression parameters. Results of the regression analysis indicate that the two regression methods yield similar results.

Some variables showed a pattern associated with nectivory categories (destructive, nondestructive, unknown) when examined using regression analysis. An analysis of variance (ANOVA) was carried out on these variables to explore how each one varied between destructive and nondestructive nectivores. A Tukey post hoc test (significance set at *P* ≤ 0.05) was used for each pairwise comparison (e.g., nondestructive nectivores versus destructive nectivores). All analyses were run using size-adjusted variables. The two most frequently applied size adjustment methods used in morphological analyses are ratios and residuals [84]. As many of the variables scale nearly isometrically with body mass, ratios and residuals should produce similar results (Table 2). For completeness, each variable was size-adjusted using residual- and ratio-based techniques. A least squares regression was used to provide residual values that are statistically uncorrelated with the independent variables. However, adding or removing species to an interspecific analysis can affect the regression slope. Thus, in addition to the residual analyses, ratio analyses were also performed (e.g., ln (cranial variable/cube root of body mass)). 

**Figure 2:**
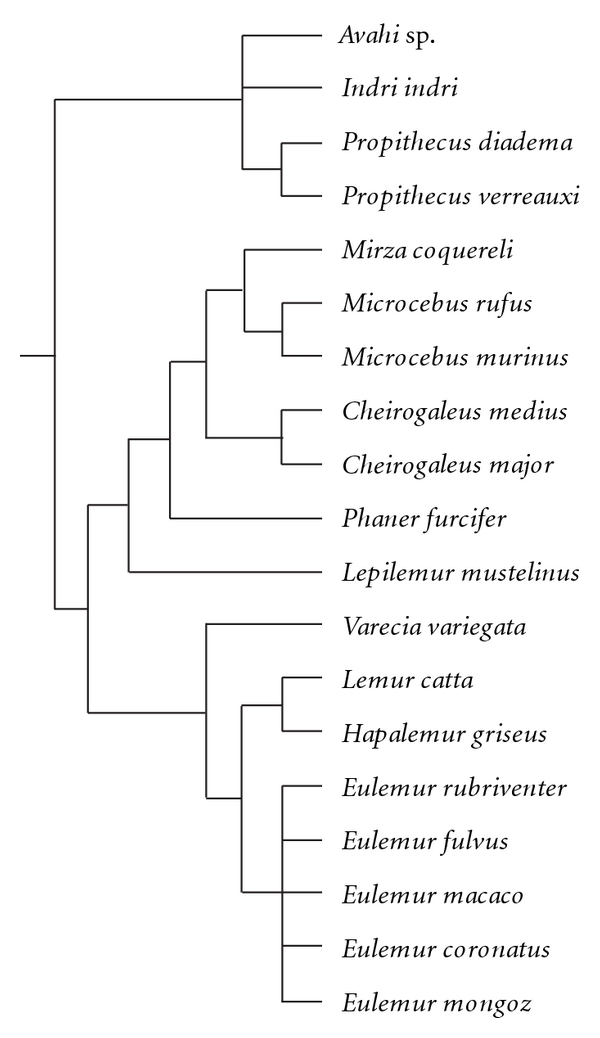
Phylogenetic branching sequence used for the taxa in this study.

Because closely related species are more likely to share ecological and anatomical characteristics than distantly related species, phylogenetic information was incorporated into the analyses. Phylogenetically independent contrasts (PICs) were calculated using PDAP : PD-TREE module [85] of Mesquite version 2.72 [86, 87]. The phylogenetic branching sequence used in this study is provided in Figure 2. The phylogeny used reflects the topology for the major clades of extant strepsirrhines generated by Horvath and colleague [88]. All branch lengths were set to one for this analysis. Contrasts were not correlated with contrast standard deviations [89].

## 3. Results

Results of the bivariate regression analyses (RMA and LS) for the entire strepsirrhine primate sample are reported in Table 2. All comparisons show high correlation coefficients (*r* = 0.87–0.96). The RMA regression analyses indicate that coronoid process height and maximum tooth row length scale isometrically with body mass. Molar area, total dentary length, total palate length, and maximum palate width scale with slight negative allometry, but the 95% confidence intervals for the slope include isometry. Total skull length, temporal muscle size, and minimum skull width scale negatively, and in no case does the confidence interval include isometry. Results from the least squares regression analyses followed a similar pattern to reduced major axis regression results: dental variables scaled with slight negative allometry, but confidence intervals include isometry mandibular variables scaled isometrically, while cranial variables scaled negatively (Table 2). When the observed regression parameters were compared to theoretical isometric slopes, results were similar (Table 2). Reduced major axis regression slopes for minimum skull length, total palate length, palate width, temporal muscle size, all mandibular variables, and maximum tooth row all scaled isometrically. Least square regression slopes for minimum skull width, total palate length, all mandibular variables, and maximum tooth row were not statistically different from a theoretical isometric slope (Table 2). 

In this study, five of the nine variables used were useful for distinguishing nondestructive nectivores from destructive nectivores. These variables are total skull length (residual: *F*(2,18) = 4.05, *P* = 0.03; ratio: *F*(2,18) = 8.96, *P* = 0.002), total palate length (residual: *F*(2,18) = 4.78, *P* = 0.02; ratio: *F*(2,18) = 7.88, *P* = 0.004), total palate width (residual: *F*(2,18) = 5.27, *P* = 0.01; ratio: *F*(2,18) = 8.03, *P* = 0.003), total dentary length (residual: *F*(2,18) = 4.35, *P* = 0.03; ratio: *F*(2,18) = 4.62, *P* = 0.03), and maximum tooth row (residual: *F*(2,18) = 3.75, *P* = 0.04; ratio: *F*(2,18) = 3.42, *P* = 0.05). These five variables, with the exception of total palate width, perhaps represent a character complex because they are all associated with muzzle elongation. Each variable is discussed individually below.

**Figure 3:**
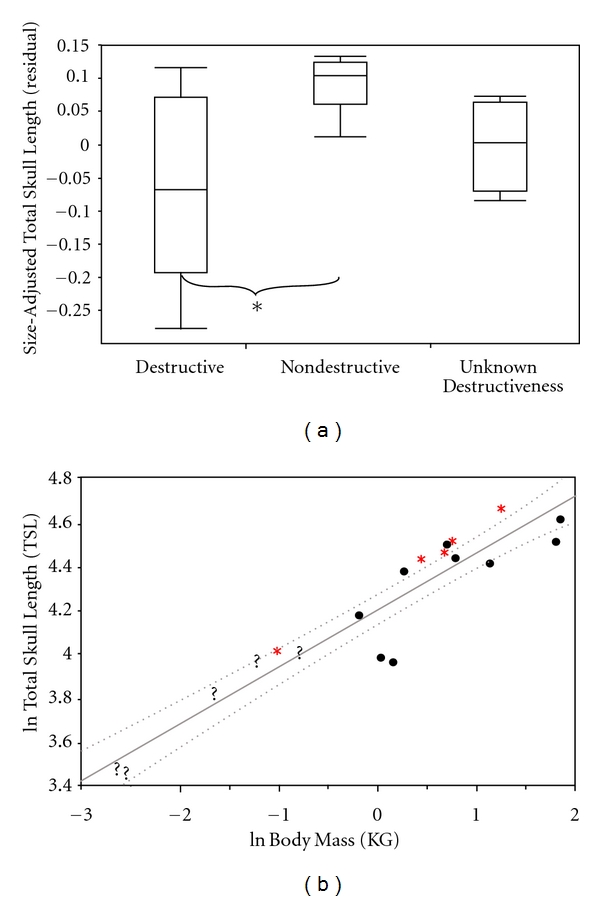
Box-and-whiskers plot illustrating differences among Malagasy strepsirrhines in total skull length (mm) of the three nectar-feeding groups (∗ < 0.05). (b) is a least squares regression of the natural log of total skull length versus the natural log of body mass (kg). This graph shows where the destructive nectar feeders (black circles), nondestructive nectar feeders (red asterisk), and nectar feeders of unknown destructiveness (black question makers) are located. This graph also includes 95% confidence bands (CI) for the slope.

Total skull length appears to be an informative variable segregating the nondestructive nectivores from destructive flower feeders (residual: *P* = 0.02, ratio: *P* = 0.02; Figure 3). Residual-based size-adjusted total skull length did not separate the nectivores with unknown feeding behavior from destructive flower feeders or nondestructive flower feeders. These results did not change when phylogeny was considered (nondestructive versus destructive, residual: *P* = 0.05; ratio: n/s). However, ratio-based size-adjusted total skull length did separate the nectivores with unknown feeding behavior from destructive flower feeders and nondestructive flower feeders (Table 3). It has been proposed that nectivorous animals should have relatively longer skulls than expected for their body size. Allometric regression analysis indicates that *V. variegata* and nondestructive nectivorous *Eulemur* species all plot above the upper 95% confidence band, suggesting that they have relatively longer skulls than animals of similar size. Two small-bodied omnivorous nectivores with unknown degrees of flower destructiveness, *C. major* and *M. coquereli*, appear to have slightly elongated crania. Destructive flower feeders *L. mustelinus*, *Avahi*, and *P. diadema* have relatively shorter crania than expected for their body size. Just as with total skull length, muzzle length in *M. murinus* plotted alongside destructive flower feeders (Figure 3). 

**Figure 4:**
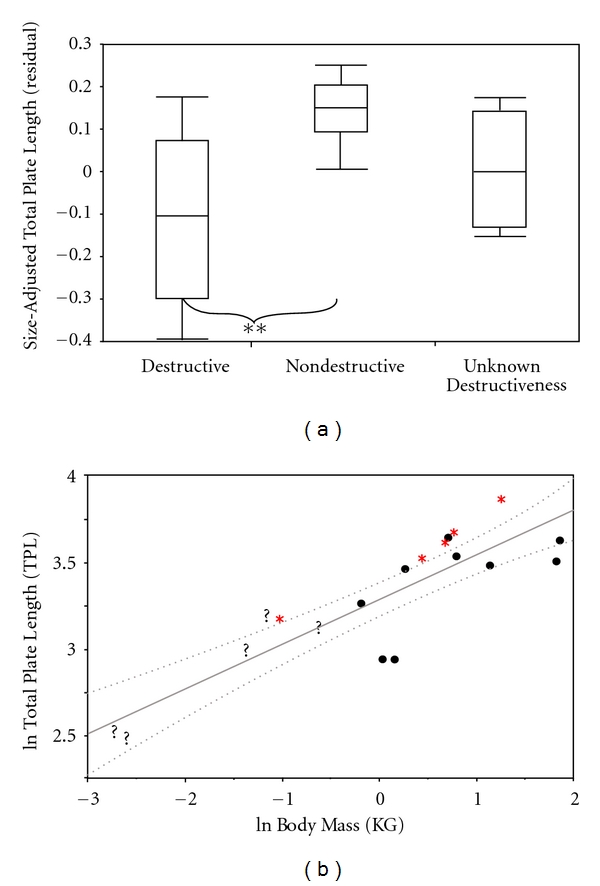
Box-and-whiskers plot illustrating differences among Malagasy strepsirrhines in total palate length (mm) of the three nectar-feeding group. (∗∗ < 0.01). (b) is a least squares regression of the natural log of total palate length versus the natural log of body mass (kg). This graph shows where the destructive nectar feeders (black circles), nondestructive nectar feeders (red asterisk), and nectar feeders of unknown destructiveness (black question makers) are located. This graph also includes 95% confidence bands (CI) for the slope.

Total palate length is a rough estimate of muzzle length. Nectivores are predicted to have long palates. Total palate length distinguishes between nondestructive and destructive flower feeders (residual: *P* = 0.01, ratio: *P* = 0.01; Figure 4). These findings did not change when phylogeny was taken under consideration (residual: *P* = 0.05, ratio: *P* = 0.03). With total palate length, the ratio-based size-adjustment method differed from residual-based analyses by showing a significant difference between nectar feeders with unknown feeding behavior and destructive nectar feeders (ratio: *P* = 0.01; Table 3). Regression analysis shows that *V. variegata*, *M. coquereli*, and nondestructive *Eulemur *species have longer muzzles than anticipated for their body size. Destructive flower feeders such as *Avahi sp., L. mustelinus, *and* P. diadema* have shorter muzzles than expected for their body size. Just as with total skull length, muzzle length of* M. murinus* clustered with that of the destructive flower feeders (Figure 4). 

**Figure 5:**
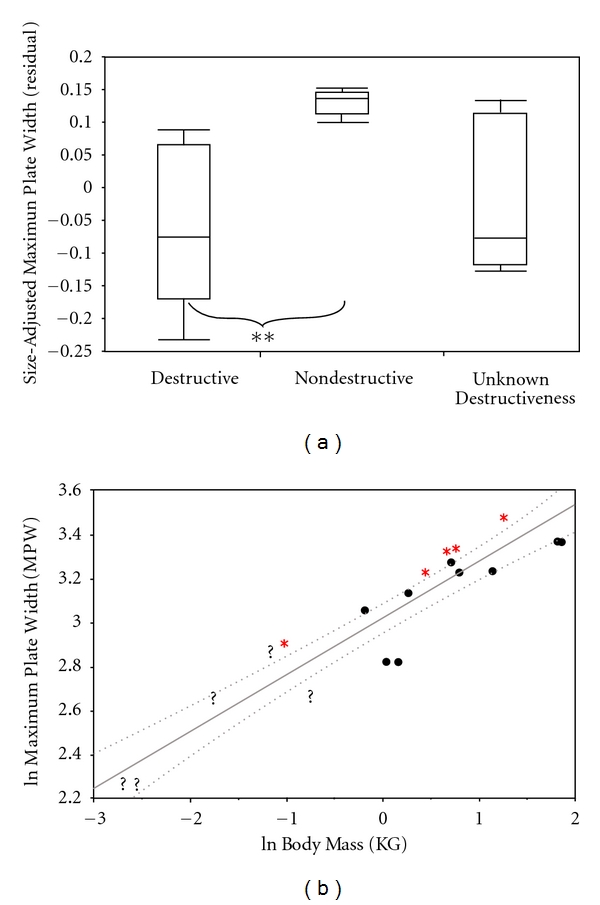
Box-and-whiskers plot illustrating differences among Malagasy strepsirrhines in maximum palate width (mm) of the three nectar-feeding groups (∗∗ < 0.01). (b) is a least squares regression of the natural log of maximum palate width (mm) versus the natural log of body mass (kg). This graph shows where the destructive nectar feeders (black circles), nondestructive nectar feeders (red asterisk), and nectar feeders of unknown destructiveness (black question makers) are located. This graph also includes 95% confidence bands (CI) for the slope.

Nectivores were predicted to have narrower palates than animals in other dietary categories. Total palate width does separate nondestructive from destructive flower feeders, but the results differ from original predictions. Ratio- and residual-based analysis yielded similar results (Table 3). Nondestructive flower feeders were found to have relatively wider palates than destructive flower feeders (residual: *P* = 0.01, ratio: 0.04; Figure 5). Correcting for phylogeny did not alter findings (residual: *P* = 0.008; ratio: *P* = 0.05). These animals have relatively wider palates than expected for their body size. Conversely, *L. mustelinus, Avahi sp.*, *P. furcifer*, and *M. murinus* all fall significantly below the lower 95% confidence bands. Other destructive nectivores fall near or on the lower 95% confidence band (Figure 5). 

**Figure 6:**
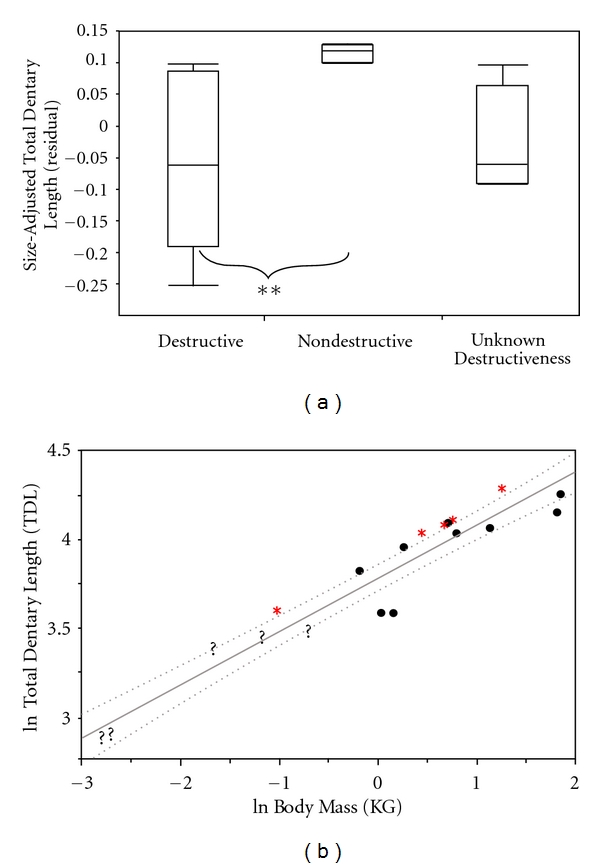
Box-and-whiskers plot illustrating differences among Malagasy strepsirrhines in total dentary length (mm) of the three nectar-feeding groups (∗∗ < 0.01). (b) is a least squares regression of the natural log of total dentary length (mm) versus the natural log of body mass (kg). This graph shows where the destructive nectar feeders (black circles), nondestructive nectar feeders (red asterisk), and nectar feeders of unknown destructiveness (black question makers) are located. This graph also includes 95% confidence bands (CI) for the slope.

Nondestructive nectivores are expected to have relatively longer dentaries than animals of similar body size. Not surprisingly, total dentary length yields similar results to those for total skull length and palate length. Nondestructive nectivores had longer dentaries than destructive nectivores (residual: *P* = 0.02, ratio: *P* = 0.02; Figure 6). Correcting for phylogeny did not alter residual findings (residual: *P* = 0.04). However, ratio size-adjusted values were different when phylogeny was considered (ratio: *P* = 0.06), but neared significance. Regression analyses revealed that *V. variegata, C. major *and most nondestructive nectivorous *Eulemur *species have long mandibular corpora compared to animals of similar size. *L. mustelinus*, *P. diadema*, *Avahi sp., M. murinus,* and *P. furcifer* plotted below the lower 95% confidence band, indicating shorter mandibles*. *The majority of these animals are destructive nectivores (Figure 6).

**Figure 7:**
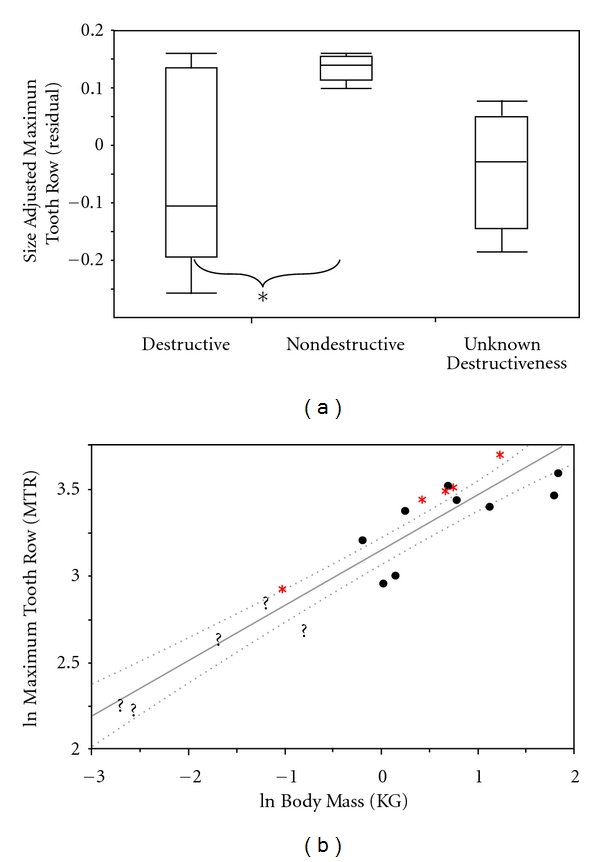
Box-and-whiskers plot illustrating differences among Malagasy strepsirrhines in maximum tooth row (mm) of the three nectar-feeding groups (∗ < 0.05). (b) is a least squares regression of the natural log of maximum tooth row (mm) versus the natural log of body mass (kg). This graph shows where the destructive nectar feeders (black circles), nondestructive nectar feeders (red asterisk), and nectar feeders of unknown destructiveness (black question makers) are located. This graph also includes 95% confidence bands (CI) for the slope.

Nectivores are expected to have an overall reduction in postcanine dentition due to a reduction in tooth use during nectar feeding. Nevertheless, in this sample, the nondestructive nectivores tend to have long tooth rows relative to body size. This feature shows a general trend of elongation among the nondestructive nectar feeders (residual: *P* = 0.05, ratio: 0.05; Figure 7). This finding is barely significant and is the opposite of the original prediction. When phylogeny was considered, the difference between nondestructive and destructive foragers lost its statistical significance using residual size-adjusted values (residual: *P* = 0.09), but not with ratio size-adjusted values (ratio = 0.05). Regression analyses highlight *V. variegata *and all nondestructive nectivorous *Eulemur *species again. These species plot above the upper 95% confidence band of a least squares regression. *P. diadema*, *L. mustelinus*, *Avahi* sp., *P. furcifer*, and *M. murinus* have a relatively shorter postcanine tooth row, plotting below the lower 95% confidence band.

### 3.1. Summary of Results

Results show that five cranial variables associated with muzzle elongation discriminate nectivores from other strepsirrhines. The nondestructive nectivores (*e.g., Varecia* and *Eulemur*) consistently show a pattern of muzzle elongation. In most cases, the omnivorous nectivores with unknown flower-feeding behavior (*e.g.*, family Cheirogaleidae) also have longer muzzles than destructive nectivores. Conversely, destructive flower feeders such as members of the families Indriidae and Lepilemuridae have a reduction in muzzle length. *P. furcifer* and *M. murinus* are two nectivores that might or might not be destructive to flowers. These two species appeared to converge with destructive flower feeders in anatomical design by having shorter muzzles than expected for their body size.

## 4. Discussion

Anatomically, nectivores are described as having an overall reduction in masticatory strength, an elongation of the cranium and muzzle, and a narrowing of the palate. In Dumont's [49] intertaxonomic analysis of nectar feeders, she pinpoints nine variables that are informative for identifying these anatomical trends. Our analyses indicate that traits associated with a decrease in masticatory strength were unsuitable for discriminating the Malagasy strepsirrhines into distinct dietary classes. By contrast, traits linked with muzzle and cranial elongation unified the nondestructive nectar feeders. The nondestructive nectar feeders in this sample were all highly frugivorous; thus, it is possible that muzzle/cranial elongation may be linked to frugivory (or to both nectivory and frugivory) rather than to nondestructive nectar feeding alone. However, because these traits sort along categories of nectivory and not along frugivore/non-frugivore lines in our sample, the expression of these traits among nectivorous strepsirrhines is more likely associated with an adaptive response to nondestructive nectar feeding rather than fruit feeding. 

The degree of dietary overlap seen in primates adds an increased level of difficulty when investigating anatomical correlates with observed foraging behaviors, given that primates are generalized in their cranial morphology relative to many orders of mammals. Malagasy strepsirrhines overlap significantly in their dietary preferences; most exploit a wide variety of food resources [90]. Traits associated with a decrease in masticatory strength are likely uninformative due to the possible overlap in resource use: although diets vary in the percentages of their constituents, it may be that the ranges of food material properties consumed by different species overlap considerably. Dumont [49] also found it impossible to discriminate based on traits related to reduction in masticatory strength: three out of five primate species used in her analysis were consistently misclassified on the basis of their anatomy. Despite the lack of specialization among the strepsirrhines of Madagascar, results for this project indicate that a distinct trend can be identified among animals that preferred particular dietary resources but this trend relates to elongation of the rostrum.

There have been no compelling biomechanical analyses of cranial shape and nectivory in primates. However, the morphological correlates of nectivory identified in this analysis possibly aid in nondestructive nectar feeding, which in turn facilitates effective cross-pollination of flowers [49, 50, 68]. The reasons being that an elongated, narrow muzzle (also reflected in a long skull) would allow penetration of the muzzle deeper into a flower corolla, with less risk of damage to surrounding flower petals and nectary.

The pattern of cranial and muzzle elongation is observed more often among the members of the genus *Eulemur* and *Varecia, *to the exclusion of the small-bodied omnivorous nectivores in many instances (*e.g., *family Cheirogaleidae). Although the small-bodied omnivorous nectivores did not perfectly parallel the large-bodied nondestructive nectivores in cranial shape, they did, however, follow a similar trend. Despite minor differences in cranial shape and size, it appears that some of the small-bodied omnivorous nectivores may in fact be nondestructive flower feeders converging anatomically because of similarities in feeding behavior. 

When dealing with size-related differences, it is important to consider how these trends correspond to observed foraging patterns. Recent work on Old World fruit bats (suborder Megachiroptera) describes differences in nectar-feeding behaviors among small- and large- bodied species [91]. Nectar-producing flowers vary in color, shape, size, and overall anatomical construction. It appears that nectivorous animals frequent different types of flowers depending on body size. Many flowers have superficial nectaries, meaning nectar is produced close to the entrance of the corolla, while others have deeper nectar chambers. Large bodied nectar feeders tend to exploit flowers that are large and sturdy in construction and have deep nectaries. On the other hand, small-bodied nectar feeders prefer smaller and more delicate flowers, many of which have superficial nectaries [36, 37, 91, 92]. Differences in flower preference among small- and large- bodied animals may have affected cranial trends in this study, as discussed below. 

**Figure 8:**
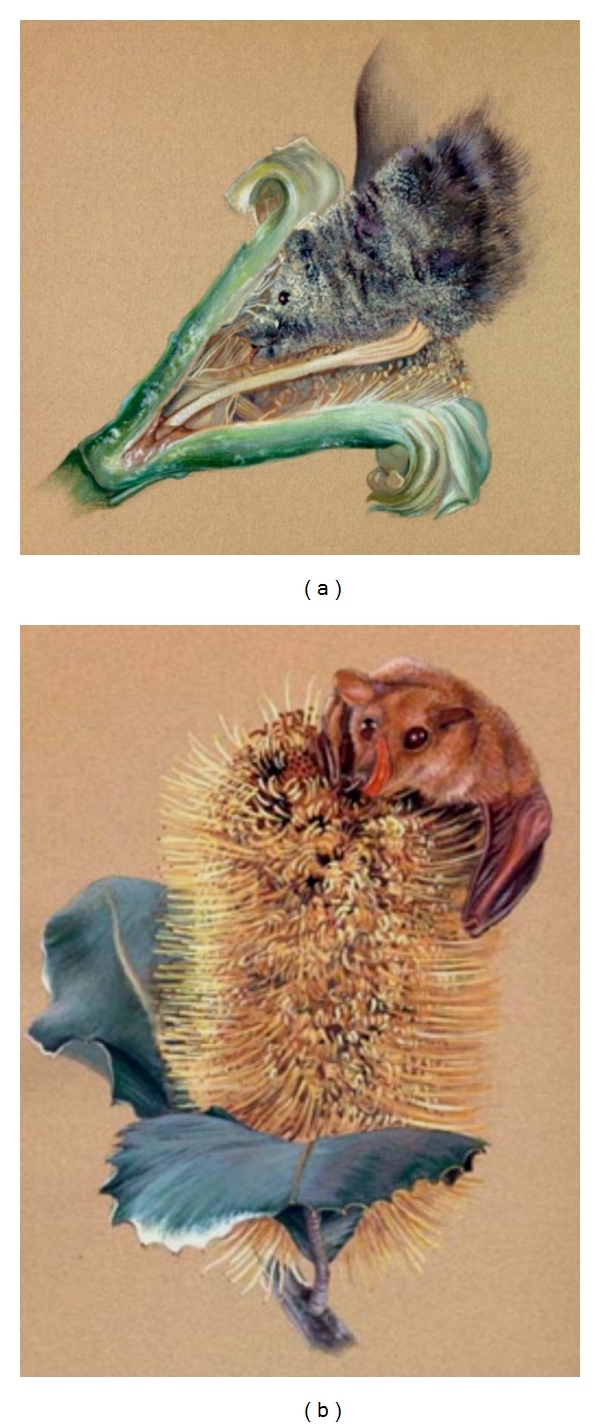
Differences in flowers and nectar-feeding technique. This drawing by Suloni Robertson was inspired by a photograph taken by Merlin [91]. (a) A long-nosed bat (*Leptonycteris curasoae*) feeding on a saguaro cactus flower. Like a lock and key, this large mammalian nectivore's head is closely matched in shape to the flower on which it is feeding. (b) A blossom bat (*Syconycteris australis*) feeding on nectar of the swamp banksias flower (*Banksia dentata*). This small-bodied nectivorous bat is feeding from superficial nectaries.

Figure 8(a) depicts a long-nosed bat (*Leptonycteris curasoae*) feeding on a saguaro cactus flower. Like a lock and key, this large nectar-feeder's head is closely matched in shape to the flower on which it is feeding. The elongation of the muzzle and cranium that is seen in nectar feeders is reported to assist these animals in reaching the base of a flower where the nectar is located [91]. When the head is withdrawn from the deep corolla of the flower, pollen collects on the muzzle and head. The pollen that sticks to the foraging animal is then transferred to other flowers. This flower-feeding technique has been observed in several species of* Varecia* and *Eulemur* [7, 36, 37, 45, 47]. It is highly probable that extreme cranial and muzzle elongation assists these two genera when they are nectar feeding on large flowers with deep nectaries.

Figure 8(b) portrays a blossom bat (*Syconycteris australis*) feeding on the nectar of the swamp banksias flower (*Banksia dentata*). This flower has superficial nectaries. *S. australis* is a small-bodied fruit bat (~15 g) whose diet is composed primarily of nectar and fruit, supplemented with other vegetation and insect prey. *S. australis* is a member of the subfamily Macroglossinae. This subfamily contains six genera whose species are much smaller in body size than other nectar-feeding bats. The chiropteran subfamily Macroglossinae and the primate family Cheirogaleidae share similarities in feeding behaviors and body size, and they appear to favor similar plant types. The Australian swamp *banksia* parallels many Malagasy flowers in overall anatomical construction. Malagasy flowers such as *Ceiba pentandra* and *Parkia madagascariensis,* which are proposed to be cross-pollinated by small-bodied omnivorous nectivores, have converged with the swamp banksias in their delicate construction, relative size dimensions, and, most importantly, their possession of superficial nectaries.

*Syconycteris australis *was included in Dumont's [49] nectar-feeding analysis, but based on its anatomy it was classified into an incorrect dietary class. This small-bodied nectivorous bat was misidentified as a frugivore because its muzzle and cranial lengths did not match the larger-bodied nectivores in Dumont's [49] sample. A similar sort of misclassification occurred among the small-bodied nectivores (classified as nectar feeders of unknown destructiveness) in this study. Since small-bodied bats and primates can exploit both smaller and larger flowers with superficial and deep nectaries (resp.), these animals might not require the same types of cranial modifications as their larger-bodied counterparts. If a small-bodied nectivore has the chance to exploit relatively large flowers with deep nectaries, exaggerated cranial and muzzle lengthening may not be needed because it may be that the entire head—not just the muzzle—fits comfortably inside the flower without any risk of damage to supporting flower structures. Thus selection for a tight fit between muzzle and flower would be relaxed in smaller nectivores.

It is important to note that although feeding technique varies among large and small animals, it is apparent that muzzle and cranial lengthening is at least advantageous to both size groups and is probably especially important for large-bodied nectivores. A long muzzle not only assists these animals in reaching nectar chambers but also houses an important nectar-feeding tool, the tongue. Nectivores are described as having long “prehensile” tongues that often have modified papillae.

Cranial and muzzle elongation characterize all but one proposed primate nectivore. In this study, *Microcebus murinus* did not plot with the nectivores; rather, it consistently grouped with the destructive flower feeders. *M. murinus *shares with destructive flower-feeding strepsirrhines a marked reduction in cranial and muzzle length and a narrowing of the palate. Based on its anatomy alone one would predict that *M. murinus *is feeding on resources that are either difficult to harvest or masticate.* M. murinus *was included by Dumont [49] but was also misclassified based on its anatomy. Rather than grouping with the frugivores (as classified by Colquhoun [47]), *M. murinus *clustered with the gumnivores. 

Narrow palates are documented in the destructive flower feeders, the gumnivore *Phaner furcifer* and* M. murinus* as a group. This finding is contrary to the original prediction linking a narrow palate with nondestructive nectar feeding. The majority of the destructive flower feeders are primarily folivorous (Table 1). Biomechanical research on the mandible shows that folivores experience midline mandibular bending during mastication [69, 93]. This loading regime likely causes loads to be transmitted to the palate (via the food), favoring evolutionary alterations in the shape and width of the palate. Unlike Dumont's finding regarding palate narrowing among mammalian nectivores, results from this project suggest that palate width is related to folivory (and possibly gumnivory, given *Phaner*) because of mid-line bending stresses in the face during mastication or gouging. Narrowing the palate may be one means of increasing bite force from the balancing-side chewing muscles while avoiding increased risk of temporomandibular joint dislocation [93–95]. This remains to be tested experimentally from the standpoint of nectivory.

Nectivory is likely not the only dietary factor acting on the distribution of cranial traits in our sample. Many of our nondestructive nectivores are primarily frugivorous, and many of our destructive nectivores are primarily folivorous. Therefore, it is important to note that the differences between fruit and leaves might influence the pattern of cranial variation observed here. Because leaves are flat, but generally tough, folivorous strepsirrhines ingest at small food sizes [96] and have relatively great jaw adductor muscle cross-sectional area [97, 98] compared to like-sized frugivorous strepsirrhines. Jaw length and skull length follow a similar trend [99]. 

If the locations of the jaw muscles are unchanged, increasing jaw/face length increases the moment arm of the bite force (decreasing bite force), and if corpus shape is unchanged, it also increases bending loads. Thus, long jaws and faces are generally not expected when bite forces are high. However, they increase linear gape at the incisors (which relates to the sizes of foods that can be ingested) relative to angular gape (which relates to stretch in the jaw muscle fibers if the attachments are unaffected). The increased need for gape and the relaxed need for powerful chewing muscles may have selected for long faces in frugivorous strepsirrhines. This is not to say that frugivory was necessarily the original adaptive context for the evolution of long faces in strepsirrhines. 

It may be that nectivory was an important part of that context, along with frugivory. Alternatively, adaptation to consuming one of those food sources might have preadapted some strepsirrhines for exploiting the other. Until we perform finer grained studies of dietary behavior and food properties in these strepsirrhines in the wild, it will be hard to distinguish between these hypotheses. Furthermore, a better understanding of the diets of the earliest primates (and the earliest strepsirrhines) would help enormously.

### 4.1. Future Avenues of Research

Despite the circumstantial evidence for Sussman and Raven's [7] hypothesis of coevolution between primates and angiosperms, there is still very little morphological evidence to support their supposition. To further test the archaic pollination hypothesis, we recommend three steps. First, detailed studies on nectar-feeding behavior need to be done on the small-bodied omnivorous nectivores of Madagascar. Second, Malagasy flower shape and dimensions need to be studied to further investigate the proposed one-to-one relationships between particular flowers and their proposed mammalian pollinators. Finally, data on fossil primate cranial and muzzle dimensions are needed to investigate whether nectar-feeding trends can be identified among these early primate forms. 

Results for this study do support a diffuse coevolutionary relationship between the Malagasy strepsirrhines and flowers they feed upon, where animals that share similar foraging behavior appear to have similarities in anatomical structures. However, it was not possible to identify whether these trends are related in a strict one-to-one coevolutionary relationship. To investigate coevolutionary trends, one needs to identify whether flowering plants, and their pollinators are working together to exclude nonadapted intruders. With the use of the data collected for this study and flower morphological data (*e.g.*, corolla depth and corolla width), it would be possible to test one-to-one coevolved relationships between flowers and the animals that pollinate them.

Despite the abundance of long-term studies detailing the foraging and dietary specializations of the extant strepsirrhines, there are still gaps that need to be filled. Further research on how Malagasy strepsirrhines exploit nectar as a resource would be tremendously informative. Small-bodied omnivorous nectivores sampled for this project appear to be exploiting nectar differently than large-bodied nectivores. Understanding how small-bodied nectivores are exploiting flowers would be informative for understanding the unique size-related trends identified in this project. Additionally, research on soft-tissue anatomical changes that are associated with nectar feeding may be informative for understanding how these animals may go about cross-pollination. Examples of soft-tissue anatomical features that appear to be linked to cross-pollinating animals include laterally oriented papillae on the tongue, laterally scaled muzzle hair, and visual sensitivity to ultraviolet light [53, 100, 101]. These specializations are seen in some Malagasy strepsirrhines; however, the information regarding these traits is largely anecdotal. Detailed documentation of these features among all proposed nondestructive nectivores is obviously needed. Finding soft-tissue anatomical changes associated with nectar feeding among the Malagasy strepsirrhines would not only strengthen the findings in this study, it would also add support for the hypothesis that Malagasy strepsirrhines act as effective and nondestructive nectar feeders.

Lastly, now that five traits have been correlated to patterns of nectivory in the strepsirrhines of Madagascar, variation in these traits could be studied in fossil primates in hopes of inferring degrees of nectivory. Although results for this project partially support Sussman and Raven's [7] hypothesis, evidences from fossil primates are needed to evaluate its temporal aspects.

## Figures and Tables

**Table 1:** Descriptive data for species in this study and mean values for nine cranial measurements associated with nondestructive nectar feeding.

Species	*N*	KG^1^	Diet^2∗^	Flower^3^	Flower feeder^4∗^	Cranial^5^						Mandibular^6^				Dental^7^	
						TMS	TSL	TPL	MPW	MSW		TDL	CPH	DD		MTR	MA
*Avahi *sp.	5	1.03	FL		D	14.7	53.4	18.9	16.6	21.4		35.5	25.6	11.1		19.1	9.7

*Hapalemur griseus*	13	0.83	FL		D	20.8	64.9	26.1	21.1	21.2		44.8	28.2	7.91		24.6	14.1

*Indri indri*	8	6.33	FL		D	25.7	100	37.8	28.7	35.1		69.9	47.3	14.8		36.1	27.9

*Cheirogaleus major*	2	0.36	OM		*Y*(ND)	22.3	55	23.8	18.2	13.9		36.3	20.4	5.96		18.3	7.3

*Cheirogaleus medius*	5	0.19	OM	*Ceiba pentandra*	*Y*(UN)	16.2	44.8	18.5	14.5	13.2		28.9	14.6	4.55		13.8	4.9
				*Rhodocolea nycteriphilla*													

*Eulemur coronatus*	10	1.30	FR		D	19.6	79.2	31.8	23	26.3		51.4	23.5	6.75		29	14.7

*Eulemur fulvus*	58	2.03	FR	*Sterculiacea *sp.	D	22.7	89.4	38.3	26.3	28.5		59.3	26.9	8.03		33.8	15.2
				*Ravensara *sp.													
				*Slonea rhodantha*													

*Eulemur macaco*	12	2.13	FR	*Parkia madagascariensis*	*Y*(ND)	23	90.9	39.4	28	29.4		60.4	27.4	9.17		33.5	12.8
				*Ravenala madagascariensis*													

*Eulemur mongoz*	4	1.55	FR	*Ceiba pentandra*	*Y*(ND)	19.7	83.6	34	25.1	29.0		55.7	25.5	7.55		30.8	12.74
				*Fernandoa madagascariensis*													
				*Hura crepitans*													
				*Combretum phaneropetalum*													
				*Sterculiacea *sp.	*Y*(ND)	24.6	86.4	37	27.6	28.0		58.6	28.5	8.92		32.8	14.4

*Eulemur rubriventer*	5	1.96	FR	*Ravensara *sp.													
				*Slonea rhodantha*													

*Lemur catta*	25	2.21	OM		D	18.6	84.1	34.4	25.2	30.7		55.7	24.4	7.6		30.8	15.4

*Lepilemur mustelinus*	23	1.17	FL	*Alluaudia ascendens*	D	15.6	52.3	18.8	16.8	18.9		35.3	17.9	5.89		19.9	10.2
				*Alluaudia procera*													
				*Delonix floribunda*	*Y*(UN)	7.95	31.7	11.9	9.47	11.9		18.5	9.73	2.98		9.26	2.5
				*Rhodocolea nycteriphilla*													

				*Ceiba pentandra*													
*Microcebus murinus*	20	0.09	OM	*Brexia madagascariensis*													
				*Rubus roridus*													
				*Uapaca *sp.													
				*Vaccinium emirnense*													

*Microcebus rufus*	4	0.07	OM		*Y*(UN)	7.22	32.3	12.6	9.45	13.7		18.3	9.2	2.37		9.55	2.9

*Mirza coquereli*	5	0.31	OM	*Parkia madagascariensis*	*Y*(UN)	13.7	52.7	23.4	17.1	18.4		31.4	15.9	4.37		17.1	6.2

*Phaner furcifer*	5	0.46	GUM	*Crateva greveana*	*Y*(UN)	27.2	54.9	22.0	14.6	19.2		31.3	13.4	5.05		14.9	3.8
				*Adansonia *sp.													

*Propithecus diadema*	5	6.10	FL		D	14.0	90.2	33.5	28.8	34.8		63.1	40.8	18.2		31.8	28.8

*Propithecus verreauxi*	21	3.10	FL		D	24.5	81.1	32.5	25.2	30.7		57.5	40.8	15.5		29.8	21.3

*Varecia variegata *	20	3.50	FR	*Ravenala madagascariensis*	*Y*(ND)	24.2	105.0	47.8	32.2	33.7		72.0	30.8	8.59		40.4	17.6
				*Labramia costata*													

^1^KG: body mass in kilograms.

^2^Primary dietary categories: FR: frugivore, FL: folivore, OM: omnivore,GUM: gumnivore.

^3^Genus (and species in some instances) of the flower that each Malagasy strepsirrhine uses most heavily.

^4^Nectivory type: destructive to other flower parts (D), nondestructive (ND), or unknown (UN).

^5^Cranial variables used in this study: TMS: temporalis muscle size, TSL: total skull length, TPL: total palate length, MPW: maximum palate width, MSW: maximum skull width.

^6^Mandibular variables used in this study: TDL: total dentary length, CPH: coronoid process height, DD: dentary depth.

^7^Dental variables used in this study: MTR: maximum tooth row length, MA: upper second molar area.

*[38, 40, 42, 43, 47, 59–66].

**Table 2:** Least squares and reduced major axis regression parameters.

	RMA	R	CI	Isometry	LS	R	CI	Isometry
Skull variables								

Minimum skull width	*y* = 0.26*X* + 3.13	0.94	.22–.31	Yes	*y* = 0.26*X* + 3.13	0.96	.19–.29	Yes
Total skull length	*y* = 0.27*X* + 4.20	0.93	.22–.32	No	*y* = 0.26*X* + 4.20	0.91	.20–.30	No
Total palate length	*y* = 0.29*X* + 3.28	0.87	.22–.36	Yes	*y* = 0.26*X* + 3.29	0.86	.17–.32	Yes
Palate width at M1	*y* = 0.27*X* + 3.01	0.93	.22–.33	Yes	*y* = 0.26*X* + 3.01	0.93	.20–.30	No
Temporal muscle size	*y* = 0.20*X* + 2.89	0.67	.09–.31	Yes	*y* = 0.20*X* + 2.89	0.46	.10–.30	No

Mandible variables								

Dentary depth M3	*y* = 0.36*X* + 1.96	0.93	.30–.44	Yes	*y* = 0.34*X* + 1.96	0.93	.27–.41	Yes
Coronoid height	*y* = 0.33*X* + 3.12	0.94	.28–.40	Yes	*y* = 0.31*X* + 3.12	0.94	.25–.37	Yes
Total dentary length	*y* = 0.30*X* + 3.77	0.95	.26–.39	Yes	*y* = 0.29*X* + 3.77	0.95	.24–.34	Yes

Dental variables								

Maximum tooth row	*y* = 0.33*X* + 3.13	0.94	.27–.39	Yes	*y* = 0.31*X* + 3.14	0.93	.25–.37	Yes
Molar Area	*y* = 0.56*X* + 1.41	0.92	.49–.72	No	*y* = 0.55*X* + 1.41	0.92	.43–.67	No

RMA: reduced major axis regression results, LS: least square regression results, CI: 95% confidence intervals, Isometry: Yes indicates that the LS or the RMA observed regression slope is not significantly different from a theoretical isometric slope. No indicates the LS or the RMA observed regression slope is significantly different from a theoretical isometric slope. Theoretical isometric slopes for MSW, TSK, TPL, TPW, TMS, DD, CH, and MTR versus body mass is 0.33. The theoretical isometric slope for molar area versus body mass is expected to be 0.66.

**Table 3:** Results of a Tukey post-hoc test (significance set at *P* < 0.05) for each pairwise comparison between feeding behavior categories.

	Nondestructive nectar feeders		Nectar feeders with unknown feeding behavior	
	Ratio	Residual	Ratio	Residual
Destructive				

Total skull length	0.02	0.02	**0.003** ^1^	**0.56**
Total palate length	0.01	0.01	**0.01**	**0.48**
Maximum palate width	0.04	0.01	0.003	0.05
Total dentary length	0.02	0.02	0.2	0.85
Maximum tooth row	0.05	0.05	0.87	0.99
Temporal muscle size	0.24	0.31	0.04	0.88

Nondestructive nectar feeders				

Total skull length			**0.07**	**0.27**
Total palate length			0.99	0.8
Maximum palate width			0.64	0.09
Total dentary length			0.57	0.12
Maximum tooth row			0.19	0.09
Temporal muscle size			0.67	0.64

^1^Bolded values indicate results that differ between ratio- and residual-based size-adjustment method.
